# Repression of the stress granule protein G3BP2 inhibits immune checkpoint molecule PD‐L1

**DOI:** 10.1002/1878-0261.12915

**Published:** 2022-01-05

**Authors:** Yanhong Zhang, Changli Yue, Anna M. Krichevsky, Igor Garkavtsev

**Affiliations:** ^1^ Department of Radiation Oncology Massachusetts General Hospital and Harvard Medical School Boston MA USA; ^2^ Department of Neurology Ann Romney Center for Neurologic Diseases Initiative for RNA Medicine Brigham and Women's Hospital and Harvard Medical School Boston MA USA; ^3^ Department of Pathology Beijing Tongren Hospital Capital Medical University Beijing China

**Keywords:** a small molecule C108, immunosuppression, PD‐L1, stress granule‐associated protein G3BP2, stress granules

## Abstract

Mounting evidence suggests that cancer stemness and immunosuppression are related, but the underlying mechanisms behind these are not clear. We previously reported that the stress granule‐associated protein G3BP2 is involved in the regulation of tumor‐initiating (stem) cells. In this study, we show that this protein also upregulates the immune checkpoint molecule PD‐L1 under conditions of stress in breast and glioblastoma cancer cells, revealing a previously unknown connection between stemness programs, stress responses, and immune checkpoint control. We also identified a significant correlation between G3BP2 and PD‐L1 co‐expression in tumor tissues from cancer patients. To assess the targetability of G3BP2, we employed a small molecule (C108) that binds G3BP2 and interferes with the stress response. Tumors treated with C108 had increased CD8 T‐cell proliferation and infiltration. Moreover, treatment of breast tumor‐bearing mice with C108 resulted in a significant survival benefit and long‐term cures. Cancer cells treated with C108 or cancer cells with genetically repressed G3BP2 had decreased PD‐L1 expression due to enhanced mRNA degradation. Our study provides a compelling mechanism linking stress granule formation and immune checkpoint program of cancer, suggesting this link may provide new opportunities for improving anticancer immunotherapy.

## Graphical abstract

Mounting evidence suggests that cancer stemness and immunosuppression are related, but the underlying mechanisms are not clear. We previously reported that the stress granule‐associated protein G3BP2 is involved in regulating tumor‐initiating (stem) cells. This study shows that G3BP2 also upregulates PD‐L1 under stress in cancer cells, revealing a previously unknown connection between stemness programs, stress responses, and immune checkpoint control.

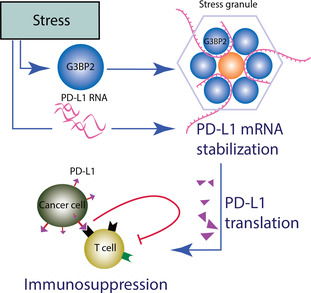

**Abbreviations**
EMT: epithelial–mesenchymal transition
G3BP2: GTPase‐activating protein (SH3 domain)‐binding protein 2
MFP: mammary fad pat
MW: molecular weights
Nanog: Nanog homeobox
Oct‐4: octamer‐binding transcription factor 4
PD‐L1: programmed death ligand 1
qRT–PCR: quantitative real‐time PCR
RIP: RNA immunoprecipitation
RRM: RNA recognition motif
SART3: squamous cell carcinoma antigen recognized by T cells 3
Scr: Scrambled
SGs: stress granules
STAT1: signal transducer and activator of transcription 1

## 1 Introduction

Cancer cells are exposed to many cell‐intrinsic and cell‐extrinsic stresses, such as nutrient and lipid deprivation, hypoxia, dysregulated proliferation, acidic extracellular pH, high levels of reactive oxygen species, and cytotoxic drugs, which collectively contribute to cancer initiation, progression, immunity, and resistance to therapy [1, 2, 3, 4, 5]. One fundamental biological program that cells initiate upon exposure to stress to limit damage and safeguard important repair machinery is the formation of stress granules (SGs) [6, 7]. SGs are dense aggregates in the cytoplasm composed of RNA and RNA‐binding proteins, including GTPase‐activating protein (SH3 domain)‐binding protein 2 (G3BP2) [8, 9, 10]. This protein coordinates the sequestration and protection of important RNAs in SGs under stress conditions. In doing so, SGs function as a decision point for untranslated mRNA to either be stored, translated, or tagged for degradation. Escaping immune surveillance, especially under stressed conditions, is essential for cancer cell survival. As such, we hypothesized that SG formation and its RNA‐binding proteins might play a role in regulating expression of immune response genes, such as programmed death ligand 1 (PD‐L1), in cancer cells. The potential interplay between SG formation and immune checkpoint programs may unveil new targets for anticancer therapy.

In previous work, we found that the G3BP2 is involved in the generation of breast tumor‐initiating cells, acting through SART3 (squamous cell carcinoma antigen recognized by T cells 3) and the pluripotency transcription factors Oct‐4 (octamer‐binding transcription factor 4) and Nanog (Nanog homeobox) [11]. A recent study has shown a strong correlation between cancer stemness and immunosuppression across 21 human tumors [12]. Similarly, a recent study by Weinberg and colleagues has shown that epithelial–mesenchymal transition—mediated by stemness transcription factors—contributes to immunosuppression in breast cancer [13]. However, the mechanisms underlying this correlation are not known.

Cancer cells often exploit immune checkpoint molecules, including PD‐L1, to suppress and evade the immune system. Targeting checkpoint pathways involved in hampering T‐cell‐mediated antitumor immunity is a major goal in cancer immunotherapy. Immune checkpoint inhibitors blocking the interaction between programmed cell death (PD‐1) and its ligand PD‐L1 have, thus far, shown the most promise in patients [14, 15]. Anti‐PD‐1 or anti‐PD‐L1 therapies are presently being evaluated in almost all types of cancer, including melanoma, lung cancer, renal cell, and Hodgkin's lymphoma [16, 17, 18]. However, major limitations, including heterogeneous expression of PD‐L1 or the presence of alternative immunosuppressive mechanisms, are thought to contribute to a lack of response in majority of patients. Therefore, a better understanding of PD‐L1 regulation should reveal additional targets for improving clinical outcomes.

PD‐L1 transcription is regulated by oncogenic RAS and MYC as well as STAT1 through RNA secondary structures present in the STAT1 (signal transducer and activator of transcription 1) 5′UTR [19, 20, 21]. However, transcriptional factors are generally not targetable and are thus not commonly used for drug discovery. Therefore, a better understanding of PD‐L1 regulation is needed to reveal additional targets for improving clinical outcomes.

## 2 Methods

### 2.1 Histologic analysis of clinical samples

Forty‐seven de‐identified clinical samples were obtained from the Department of Pathology of Beijing Tongren Hospital, China. The protocol for this research has been approved by the MGH Institutional Review Board (2019P003386). Tissue sections were stained for G3BP2 (rabbit anti‐G3BP2; Abcam, Cambridge, MA, USA, 1 : 500) and PD‐L1 (mouse anti‐PD‐L1; BioLegend, San Diego, CA, USA, 1 : 50) by immunofluorescence assay (the more details are presented in immunofluorescence method).

### 2.2 Cancer cells and their modifications

The pLKO.1‐puro cloning vector with G3BP2 (shG3BP2) together with VSVG and psPAX2 plasmids and FuGENE reagent (Promega, Madison, WI, USA) was used for transfection of 293T cells. We generated stable cancer cell lines with diminished levels of G3BP2, using lentiviral‐mediated delivery of mouse sh2G3BP2: (gtaccgggaataaagctcccgagtatttctcgagaaatactcgggagctttattcttttttg) sh3G3BP2, (ccggttcgaggagaagtacgtttaactcgagttaaacgtacttctcctcgaatttttg) cloned in pLKO.1‐puro lentiviral vector. G3BP2 downregulation was confirmed by using both quantitative real‐time PCR (qRT–PCR) and western blotting.

We cloned the full length of G3BP2 cDNA fragment in a retroviral vector under the LTR promoter for overexpression experiments. We used mouse RNA from different organs and forward (cgggatccgccaccatggttatggagaagcctagtcccct) and reverse (gcgcgtcgac ttcaacgacgctgtcctgtgaagcga) G3BP2 primers for cDNA synthesis. The full length of mouse cDNA G3BP2 was cloned into BamHI/SalI sites of the pBaBe retroviral vector, and the structure of the cloned gene was confirmed by sequencing analysis.

### 2.3 Western blotting

Lysates from CT2A, MCa‐PSTC, and 4T1 cells were prepared in lysis buffer containing Tris/HCl 50 mm/pH 7.4; NaCl 150 mm; NP‐40 1%; SDS 0.1%; Na/deoxycholate 0.5%; EDTA 1 mm; plus 1% phosphatase inhibitor cocktails I and II (Sigma‐Aldrich, St Louis, MO, USA); and 1% protease inhibitor cocktail (Roche, Indianapolis, IN, USA). Equal amounts of protein (30 μg/sample) were resolved by SDS/PAGE, transferred onto nitrocellulose membranes, and immunoblotted using the following primary antibodies: anti‐PD‐L1 (Abcam, 1 : 1000), anti‐G3BP2 (Abcam, 1 : 1000), and β‐actin (Sigma; St Louis, MO, USA, 1 : 5000). Immunodetection was performed by incubation with HRP‐conjugated species‐specific antibodies (Cell Signaling Technology, Danvers, MA, USA, 1 : 5000), followed by chemiluminescence detection (Perkin Elmer, Waltham, MA, USA).

### 2.4 Quantitative real‐time PCR

Total RNA was extracted using the Qiagen RNeasy Mini Kit (Santa Clarita, CA, USA). Total RNA (1 µg) was reverse‐transcribed using the Reverse Transcription System (InScript, Bio‐Rad, Hercules, CA, USA). qRT–PCR was performed using SYBR Green Reagents (Bio‐Rad) with the following cycling conditions: 95 °C for 10 min, followed by 40 cycles at 95 °C for 15 s, 60 °C for 60 s, and 72 °C for 60 s. We used next primers for PD‐L1 detection 5′‐atgcccgcttccagatcata‐3′ (forward) and 5′‐tcgatttttgccttggggtg‐3′ (reverse) and primers for GAPDH amplification 5′‐aggtcggtgtgaacggatttg‐3′ (forward) and 5′‐tgtagaccatgtagttgaggtca‐3′ (reverse).

### 2.5 Immunofluorescence assay

MCa‐PSTC and CT2A cells were fixed with 4% paraformaldehyde. Cells were permeabilized with PBS containing 0.1% Triton X‐100 for 10 min and blocked in 5% NDS and 0.3% Triton X‐100 in PBS for 1 h. The cells were then incubated overnight at 4 °C with primary antibodies (rabbit anti‐PD‐L1, 1 : 200) and further incubated with secondary antibodies (goat anti‐rabbit, 1 : 200). The nuclei were stained with DAPI (1 : 1000). The cellular staining was viewed and captured on a scanning confocal microscope.

The breast cancer tissues (47 samples) were embedded with paraffin, and slides (4 μm) were deparaffinized in xylenes and hydrated through graded alcohols. The sections were boiled in antigen unmasking solution (pH 6.0) at 97 °C for 20 min and incubated with 0.3% H_2_O_2_ for 20 min. After blocking with 5% normal donkey serum (NDS), the slides were incubated with G3BP2 (rabbit anti‐G3BP2; Abcam, 1 : 500) and PD‐L1 (mouse anti‐PD‐L1; BioLegend, 1 : 50) antibodies overnight at 4 °C. The next day incubation with secondary antibodies (anti‐rabbit, 1 : 200, and anti‐mouse, 1 : 200) was performed at room temperature for 1 h. The nuclei were stained with DAPI (1 : 1000). RGB images of the tissue were acquired using a Hamamatsu NanoZoomer S360 Slide Scanner (Hamamatsu, Japan).

### 2.6 RNA immunoprecipitation assay

The direct interactions between PD‐L1 mRNA and G3BP2 protein were analyzed using the RNA‐Binding Protein Immunoprecipitation Kit (Millipore, Burlington, MA, USA) according to the manufacturer's instructions. The IgG control and anti‐G3BP2 antibodies were used for RNA immunoprecipitation (RIP) experiments. Total RNA was isolated from MCa‐PSTC and CT2A cells, and qRT–PCR was carried out using the method specified in the above section. To identify the interactive part of the mRNA, we used PD‐L1 RNA transcripts obtained from T7 promoter‐PD‐L1 DNA fragments with T7 RNA polymerase. We used 30 μg of RNA products and performed RIP assays with IgG control and anti‐G3BP2 antibodies. RNA was measured by qRT–PCR using primers [CDS fragment: 5′‐gctccaaaggacttgtacgtg‐3′ (forward) and 5′‐tgatctgaagggcagcatttc‐3′ (reverse) and 3′UTR fragment: 5′‐ctgagggagagaaccaagaaag‐3′ (forward) and 5′‐ccctcaatccatttcccaaga‐3′ (reverse)].

### 2.7 Animal studies

All animal experiment procedures were performed following the Guidelines of Public Health Service Policy on Humane Care of Laboratory Animals and approved by the Institutional Animal Care and Use Committee (IACUC) in Massachusetts General Hospital (MGH). Mice were bred and housed in the Edwin L. Steele Laboratories Animal Facility in MGH. For the MCa‐PSTC breast cancer model, 5 × 10^5^ cells were injected into the mammary fad pat (MFP) of 6‐ to 8‐week‐old female FVB/N mice. When the tumor size reached 5 mm in diameter, the tumor‐bearing mice were treated twice per day with C108 compound (30 mg·kg^−1^ in DMSO) or DMSO (vehicle‐treated) for 26 days. For the 4T1 breast cancer model, 4T1 or 4T1‐shG3BP2 (1 × 10^5^) cells were injected into the MFP of 6‐week‐old female BALB/c mice. When the tumor size reached 3 mm in diameter, mice were treated with vehicle or compound C108 (30 mg·kg^−1^) twice per day for 10–12 consecutive days.

For the survival assay, BALB/c mice bearing 4T1 tumors (1 × 10^4^ cells injected into the MFP) were used for treatment. When tumors reached a size of 3 mm (4T1), animals were randomized into three groups: control, anti‐PD‐L1, and C108 treatment groups. Pharmacokinetic studies indicate that after intraperitoneal dosing of C108 at 5 mg·kg^−1^, an average C_MAX_ of 132 ± 46 ng·mL^−1^ was reached at 0.25 h postdose with an average half‐life of 4 h. Mice were treated with DMSO or anti‐PD‐L1 antibody (Bio X Cell, BE0101, West Lebanon, NH, USA) at a dose of 200 µg per mouse every three days or with C108 (30 mg·kg^−1^ in DMSO) twice per day. We resected the primary tumors when the largest tumor reached 10 mm in diameter and continued treatment 10 days postsurgery. We used the Kaplan–Meier survival analysis to determine the effects of C108 compound and anti‐PD‐L1 antibodies on mice survival.

For 4T1 long‐term survivor rechallenge experiment, we selected the same age BALB/c mice as naïve group matched with rechallenged group and injected 1 × 10^4^ 4T1 cells into the MFP. We resected the primary tumors when the largest tumor reached 10 mm in diameter and observed the survival time in the two groups.

### 2.8 Immunohistochemistry

Tissues were embedded in paraffin. Sections (4 μm) were deparaffinized in xylenes and hydrated through graded alcohols. Antigen retrieval was performed in antigen unmasking solution at 97 °C for 20 min. Slides were treated with 0.3% H_2_O_2_ peroxidase for 20 min and blocked with 5% NDS for 1h at room temperature. PD‐L1 antibody (1 : 50) and CD45 (1 : 100) antibody were incubated overnight at 4 °C, washed, and then incubated for 30 min at 37 °C with an anti‐rat HRP‐coupled secondary antibody. Images were analyzed using image‐pro plus 6 (Media Cybernetics, Silver Spring, MD, USA). After image background correction, the program determined the number of positive pixels and the integrated optical density. The total area of each tissue section was determined using automated segmentation (Otsu's method) in FIJI. The number of positive pixels was divided by the tissue area to get the fraction of positive pixels.

### 2.9 Flow cytometry

Whole tumors were harvested, weighed, minced, and digested into a single‐cell suspension using collagenase (1.5 mg·mL^−1^) and hyaluronidase (1.5 mg·mL^−1^) and DNase (20 μg·mL^−1^) in HBSS media at 37 °C for 20 min. The cell suspension was then passed through a 70 μm mesh. Cell pellets were collected by centrifugation (5 min, 4 °C, at 300 ***g***). Cells FC were blocked using CD16/CD32 antibody [15 min in FACS buffer (2% FBS in PBS) at 4 °C]. Cells were then washed and incubated with anti‐mouse antibodies for extracellular staining for 20 min at 4 °C with the following dilutions (TCRb: 1 : 200, CD4: 1 : 200, CD8: 1 : 200, dilution). Finally, cells were sorted using LSRII and the data were analyzed using flowjo software (Ashland, OR, USA).

### 2.10 Statistical analysis

For statistical analysis, jmp statistical analysis software was used (SAS Institute, Cary, NC, USA). Two‐tailed *t*‐tests were used to compare data between two groups. We considered a *P*‐value less than 0.05 to be statistically significant (*P < 0.05, **P < 0.01, ***P < 0.001, ****P < 0.0001). Statistical significance for the colocalization analyses was tested using the nonparametric Spearman's *t*‐test. Correlation analysis for G3BP2 and PD‐L1 staining in 47 clinical breast samples was then performed using the Coloc 2 plugin in fiji [22]. Images were split into G3BP2, PD‐L1, and DAPI channels, and an R01 was placed to select the tumor area using the DAPI channel before running the analysis. This was repeated for each of the 47 tissue samples (11 basal‐like, 13 Her‐2‐enriched, and 23 luminal‐like). The Coloc 2 output includes Pearson's correlation coefficients and associated Spearman's *t*‐test statistics.

## 3 Results

### 3.1 Stress increases PD‐L1 expression in cancer cells

**Fig. 1:**
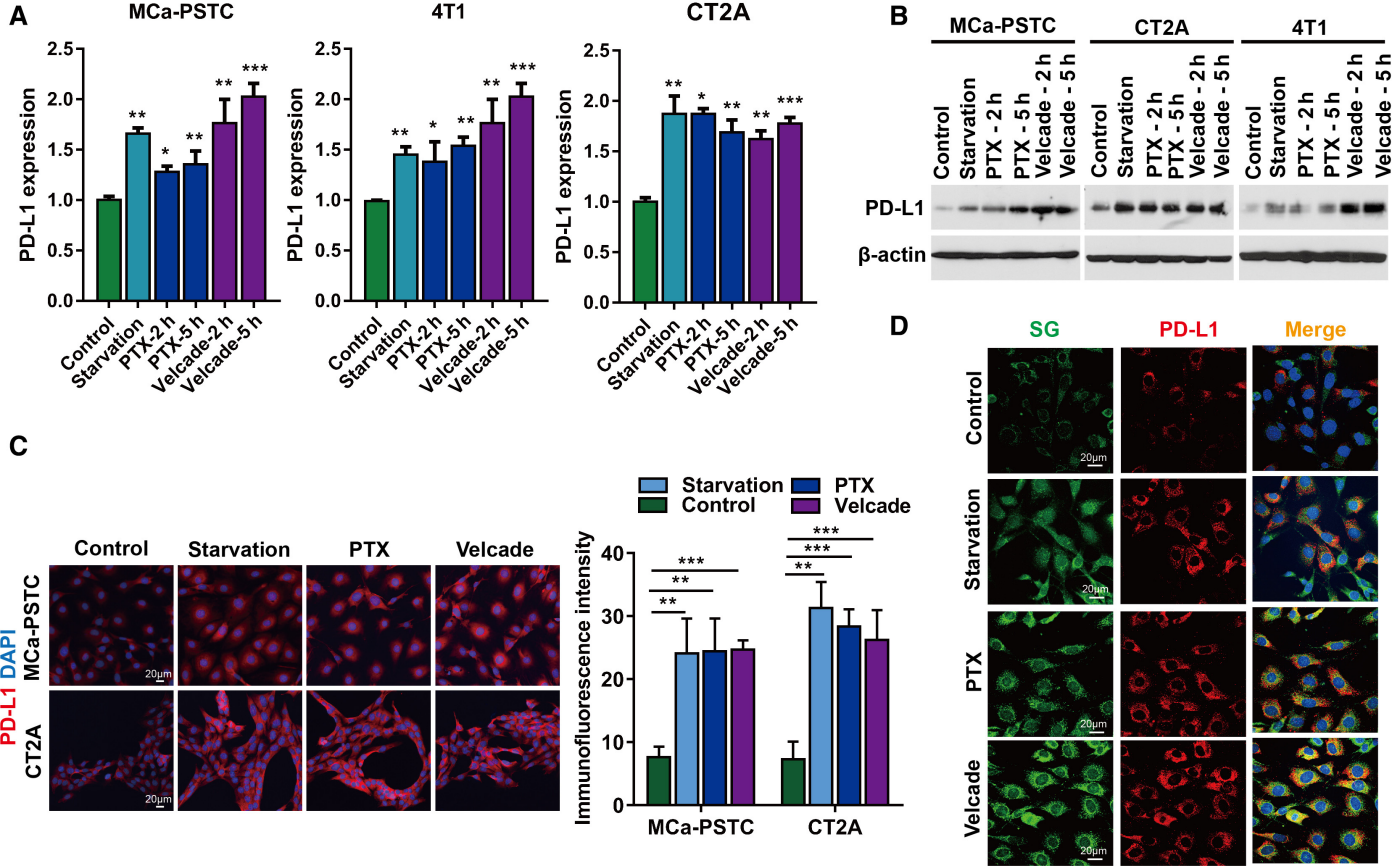
Cancer cells show elevated levels of PD‐L1 protein in response to stress. (A) qRT–PCR analysis of MCa‐PSTC, CT2A, and 4T1 cells after starvation for 16 h and treatment with paclitaxel (PTX, 1 μm) or velcade (1 μm) for 2 or 5 h. Data are mean ± SD of three experiments. *P < 0.05, **P < 0.01, ***P < 0.001. (B) Western blotting with lysates from MCa‐PSTC, CT2A, and 4T1 cells and antibodies raised against PD‐L1 protein show that PD‐L1 protein increases in response to stresses, including starvation (16 h), paclitaxel (PTX, 1 μm, 2 or 5 h), and velcade (1 μm, 2 or 5 h). β‐actin was used as a loading control. Data are mean ± SD, *P < 0.05, **P < 0.01, ***P < 0.001, ****P < 0.0001. (C) Immunofluorescent staining of PD‐L1 (red) on stressed cancer cells imaged by confocal microscopy. DAPI (blue) was used for nucleus staining and quantitative analysis of immunostaining experiments. Data are mean ± SD, **P < 0.01 and ***P < 0.001. Scale bar = 20 µm. (D) MCa‐PSTC cells were stained with stress granule marker (PAPB) and PD‐L1 by immunofluorescence microscopy. Representative images show co‐expression between PD‐L1 (red) and PAPB (green) under different stress conditions. Scale bar = 20 µm.

To interrogate the potential relationships between cancer cell stress responses and immune evasion/suppression, we exposed cancer cells to chemical stresses or starvation and studied the expression of PD‐L1 protein. In this study, we analyzed one mouse glioblastoma (CT2A) and two mouse breast cancer lines with different metastatic potentials (MCa‐PSTC and 4T1). MCa‐PSTC is a cell line established from spontaneous mammary carcinomas arising in female MMTV‐PyVT/FVB transgenic mice in Steele Laboratories (Massachusetts General Hospital, Boston, MA, USA). 4T1 (ATCC, CRL‐2539™) is a highly metastatic murine mammary carcinoma [23] and is more aggressive than MCa‐PSTC. To identify the programs common to different cancers, we analyzed mouse MCa‐PSTC (lower metastatic murine mammary carcinoma), 4T1 (highly metastatic murine mammary carcinoma), and CT2A (glioblastoma) cells. These cells have different growth characteristics and model highly distinct pathophysiologies, but may share biological mechanisms for coping with stress. We starved cancer cells or stressed them with chemotherapy, and then performed qRT–PCR, western blot, and immunostaining with antibodies against PD‐L1 protein (Fig. 1A–C). The results indicated that PD‐L1 level is increased in starved or chemically stressed cancer cells. To confirm the effects of stress on PD‐L1 expression *in vivo*, we treat animals with paclitaxel (Fig. S1) and dissect breast cancer tumors. Tumors from treated and untreated animals were stained with anti‐PD‐L1 antibodies. Consistent with the *in vitro* results, the PD‐L1 levels were increased in the chemotherapy group compared with the control group.

During stress, cells can modulate the translation and stability of critical mRNAs by sequestering them to stress granules (SG) [4, 8]. We next explored whether SG involvement regulated PD‐L1 expression. Different stress conditions (starvation, paclitaxel, and velcade treatments) promoted the SG formation in MCa‐PSTC cells (Fig. 1D). Moreover, MCa‐PSTC cells showed higher co‐regulation of SG formation and PD‐L1 under stresses compared with the nonstressed control group (Fig. 1D). Taken together, these results demonstrated that SG formation may play a critical role in the upregulation of PD‐L1.

### 3.2 PD‐L1 protein level in cancer cells is dependent on G3BP2

**Fig. 2:**
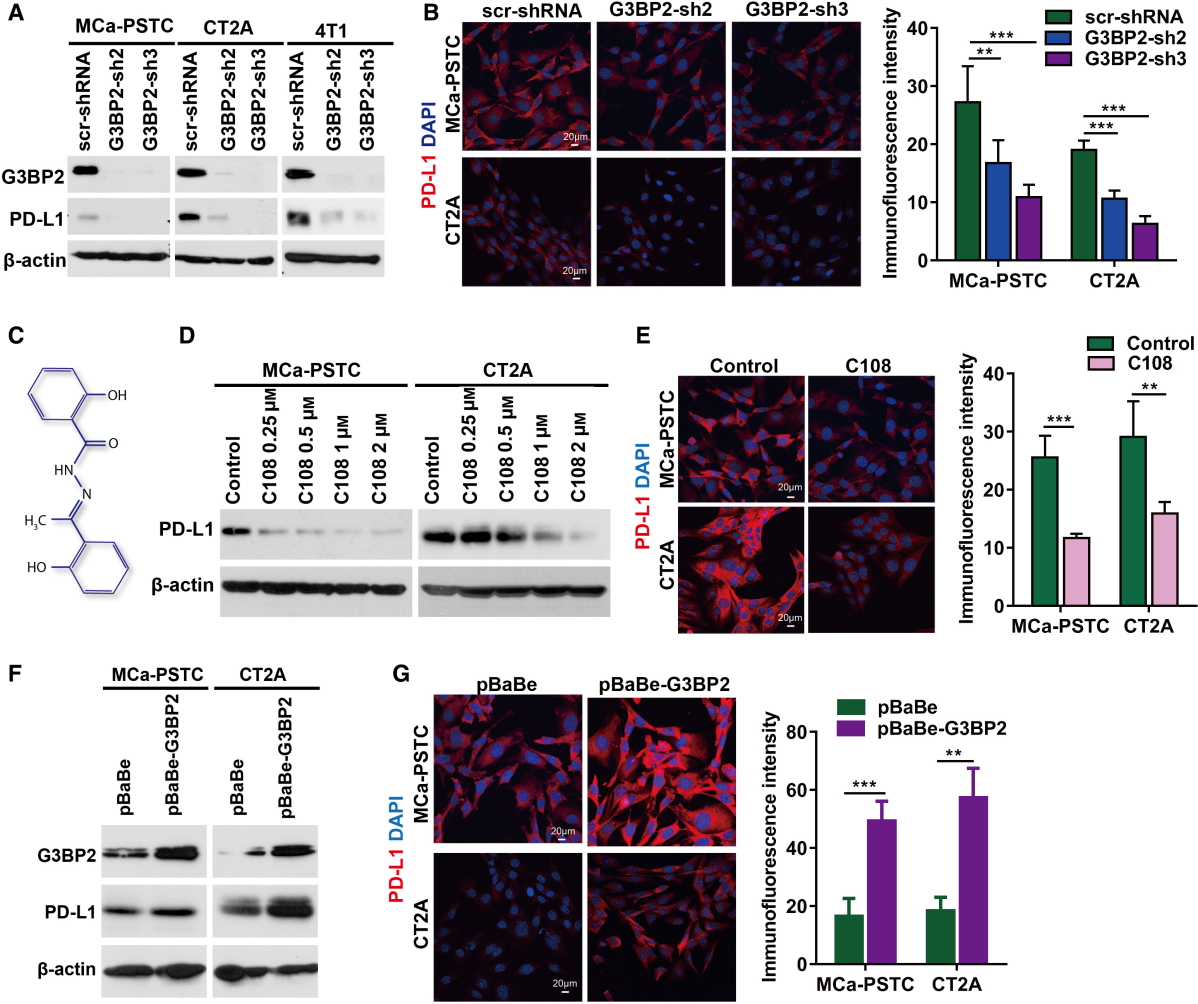
PD‐L1 protein level in cancer cells is dependent on G3BP2. (A) Western blotting analysis of G3BP2 and PD‐L1 expression in MCa‐PSTC, CT2A, and 4T1 cells with two G3BP2 shRNAs. Decreased G3BP2 expression leads to PD‐L1 repression. (B) Immunostaining for PD‐L1 protein (red) and DAPI (blue) for the cells with two different G3BP2 shRNAs and quantitative analysis of immunostaining experiments. Data are mean ± SD; **P < 0.01 and ***P < 0.001. Repression of G3BP2 decreases PD‐L1 level. (C) Structure of compound C108 (2‐hydroxy‐*N*′‐[1‐(2‐hydroxyphenyl)ethylidene]benzo‐hydrazide) (B) MCa‐PSTC and CT2A cells were starved for 16 h and treated with compound C108 (1 μm) for 24 h and measured the PD‐L1 expression by immunostaining assay. **P < 0.01. β‐actin was used as a loading control. Scale bar = 20 µm. (E) Immunofluorescent staining of PD‐L1 (red) on treated with C108 cancer cells imaged by confocal microscopy. DAPI (blue) was used for nucleus staining and quantitative analysis of immunostaining experiments. Data are mean ± SD; scale bar = 20 µm. (F) MCa‐PSTC and CT2A cells were infected with pBaBe as a control or with pBaBe‐G3BP2 retroviruses. Overexpression of G3BP2 was detected by western blotting. Increased G3BP2 levels lead to accumulation of PD‐L1 protein. β‐actin was used as a loading control. (G) Immunostaining for PD‐L1 protein (red) and DAPI (blue) for the cells with overexpressed G3BP2 and quantitative analysis of immunostaining experiments. Data are mean ± SD; scale bar = 20 µm.

Because G3BP2 is one of the key proteins forming the core of SGs [9, 10, 11], we hypothesized that PD‐L1 and G3BP2 might be co‐regulated. To determine whether upregulation of PD‐L1 protein by stress depends on G3BP2, we genetically suppressed G3BP2 using two different lentiviral shRNAs and assessed protein expression by western blot. G3BP2 suppression inhibited the stress‐induced expression of PD‐L1 in MCa‐PSTC, CT2A, and 4T1 cancer cells (Fig. 2A). We further confirmed that G3BP2 suppression decreases PD‐L1 protein in cancer cells using immunostaining (Fig. 2B).

To determine whether pharmacological blockade of G3BP2 can affect stress‐induced PD‐L1 expression, we starved MCa‐PSTC, CT2A, and 4T1 cells and treated with C108, a small molecule inhibitor of G3BP2 (Fig. 2C) [11]. Treatment with C108 for 24 h decreased PD‐L1 expression in a concentration‐dependent manner (Fig. 2D,E). We next checked whether the overexpression of G3BP2 could increase PD‐L1 expression (Fig. 2F,G). Our data indicated that the increased level of G3BP2 leads to the increased level of PD‐L1 in cells. Together, these results show that G3BP2 directly controls PD‐L1 level.

### 3.3 Co‐expression of G3BP2 and PD‐L1 in clinical tumor tissues

**Fig. 3:**
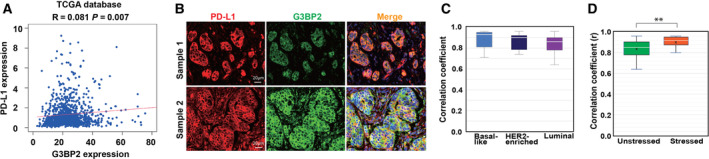
Co‐expression of G3BP2 and PD‐L1 in breast cancer tissues. (A) The co‐expression of G3BP2 and PD‐L genes in 1109 breast cancer samples from TCGA database. Pearson's correlation coefficients and associated Spearman's *t*‐test were used for statistics. (B) Human breast cancer specimens were stained for G3BP2 and PD‐L1 co‐expression by immunofluorescence microscopy. Representative images show correspondence between PD‐L1 (red) and G3BP2 (green) staining patterns. Images from two ER+ patients are shown. Scale bar = 20 µm. (C) The linear regression analysis of PD‐L1 and G3BP2 co‐expression was detected by immunostaining in breast tumor tissues (positive correlation *R* ^2^ = 0.8–0.9). Data are mean ± SD. (D) Colocalization results under different stress conditions of breast cancer patients. Stressed group: patients receiving neoadjuvant chemotherapy before surgery; nonstressed group: patients with treatment‐naïve tumors. Data are mean ± SD, statistical significance for the analyses was tested using the nonparametric Spearman's *t*‐test (**P < 0.01).

To determine whether our cell‐based data recapitulate the cancer features, we first examined the co‐expression of G3BP2 and PD‐L1 genes in 1109 patient tissues diagnosed with breast cancer from TCGA database. The result indicated that G3BP2 and PD‐L1 mRNAs co‐expressed frequently together (*R* = 0.081, *P* = 0.007; Fig. 3A). To confirm these data, we checked for correlation between G3BP2 and PD‐L1 proteins in 47 patient samples representing different breast cancer subtypes and found that G3BP2 and PD‐L1 were positively correlated (overall *R* ^2^ = 0.8–0.9, *P* < 0.0001; Fig. 3B,C). These data suggested that G3BP2 and PD‐L1 proteins correlate much stronger than the corresponding mRNAs. Additional post‐transcriptional mechanisms might be regulating G3BP2 expression. We then further focused the analysis on patients treated with chemotherapy. Patients who had received neoadjuvant chemotherapies, including adriamycin, paclitaxel, cyclophosphamide, and trastuzumab before surgery (stressed group), were compared to treatment‐naïve patients (nonstressed group). The results show that colocalization of G3BP2 and PD‐L1 was higher in the stressed group than the nonstressed group (Fig. 3D), supporting the hypothesis that G3BP2 and PD‐L1 are co‐upregulated in breast tumors exposed to chemical stress.

### 3.4 Genetic and pharmacological repression of G3BP2 leads to PD‐L1 suppression *in vivo*

**Fig. 4:**
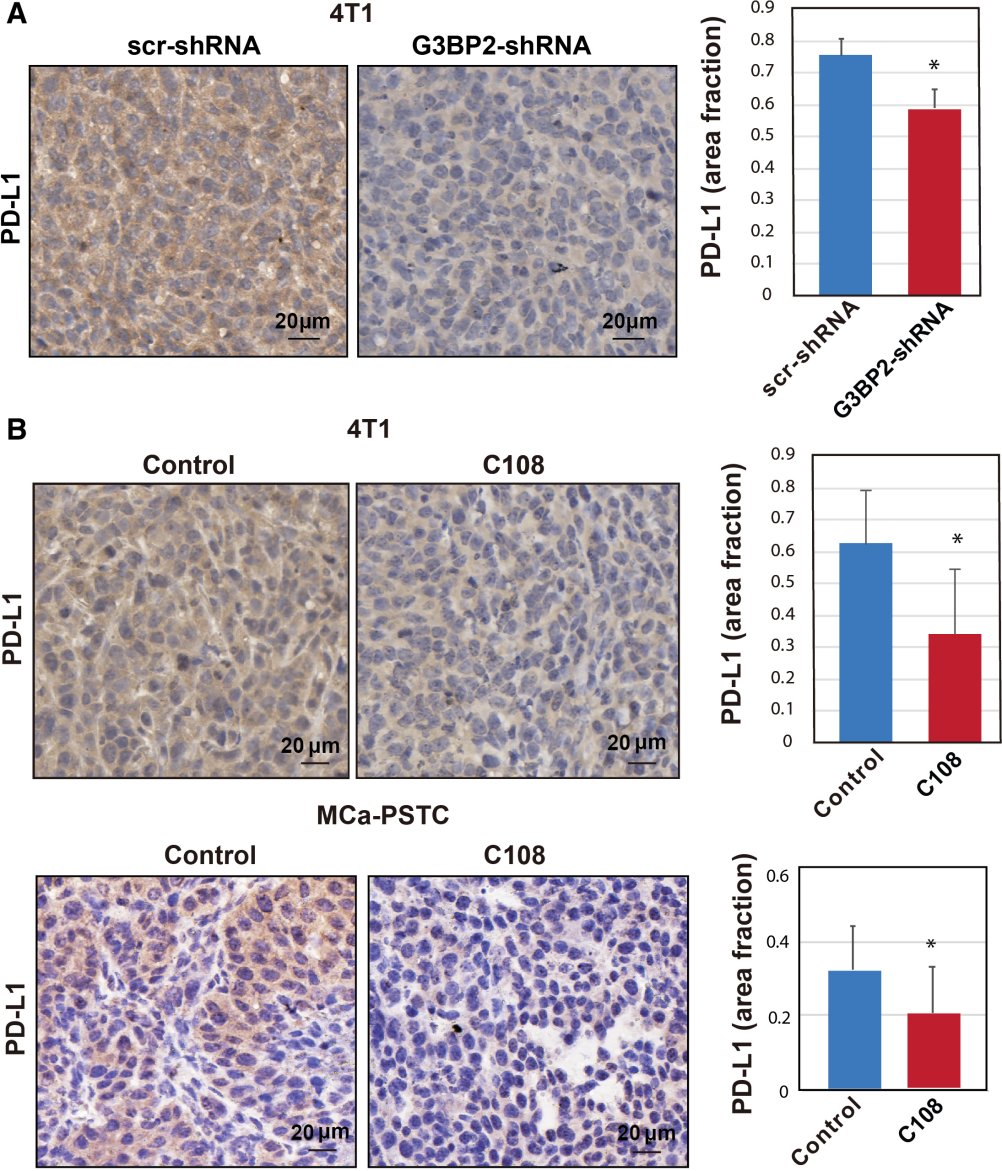
Repression of PD‐L1 expression *in vivo* after C108 treatment in 4T1 and MCa‐PSTC tumors. (A) Scr‐shRNA or shG3BP2 4T1 cells were injected into the mammary fat pad of BALB/c mice. Paraffin‐embedded sections of tumor tissues were used for immunohistochemistry assay with PD‐L1 antibody (*n* = 9 tumors per group). Quantitative analysis of PD‐L1‐positive cells in tumors with different expression of the G3BP2. Data are mean ± SD; *P < 0.05. Scale bar = 20 µm. (B) Representative images and quantitative analysis of PD‐L1‐positive cells in C108‐treated and C108‐untreated tumors. Paraffin‐embedded sections of tumor tissues were used for immunohistochemistry with a PD‐L1 antibody. Tissues from C108‐treated and C108‐untreated tumors show decreased PD‐L1 with C108 treatment (*n* = 9 tumors per group). Data are mean ± SD; *P < 0.05. Scale bar = 20 µm.

Encouraged by the *in vitro* analyses and retrospective patient tissue results, we next tested whether reduced expression of G3BP2 would decrease PD‐L1 levels in orthotopic murine tumors *in vivo*. We performed experiments with shRNA‐mediated suppression of G3BP2 in 4T1 cells and measured PD‐L1 levels using immunohistochemistry in Scrambled (Scr) and shG3BP2 tumors (Fig. 4A). Suppression of G3BP2 resulted in decrease PD‐L1 expression in primary tumors (Fig. 4A). These results confirm our *in vitro* data that G3BP2 is an important regulator of PD‐L1.

To confirm that PD‐L1 protein expression is modulated by G3BP2 *in vivo*, we treated animals bearing orthotopic MCa‐PSTC and 4T1 tumors with G3BP2 binding compound (C108) and measured PD‐L1 levels using immunohistochemistry. MCa‐PSTC and 4T1 cells were grown in the mammary fat pad of immunocompetent female FVB/N or BALB/c mice. Beginning on day 7 after implantation, when tumors were ~ 3.0 mm in diameter, the mice received daily intraperitoneal injections of C108 (or DMSO as a vehicle). C108 was well‐tolerated, as the animals showed no signs of weight loss, or abnormal appearance or behavior. As expected, G3BP2 blockade with C108 decreased PD‐L1 protein in tumors (Fig. 4B). Taken together, these data show that pharmacological or genetic suppression of G3BP2 protein decreases PD‐L1 expression *in vivo*.

### 3.5 C108 compound increases tumor‐infiltrating hematopoietic‐derived immune cells in primary tumors

**Fig. 5:**
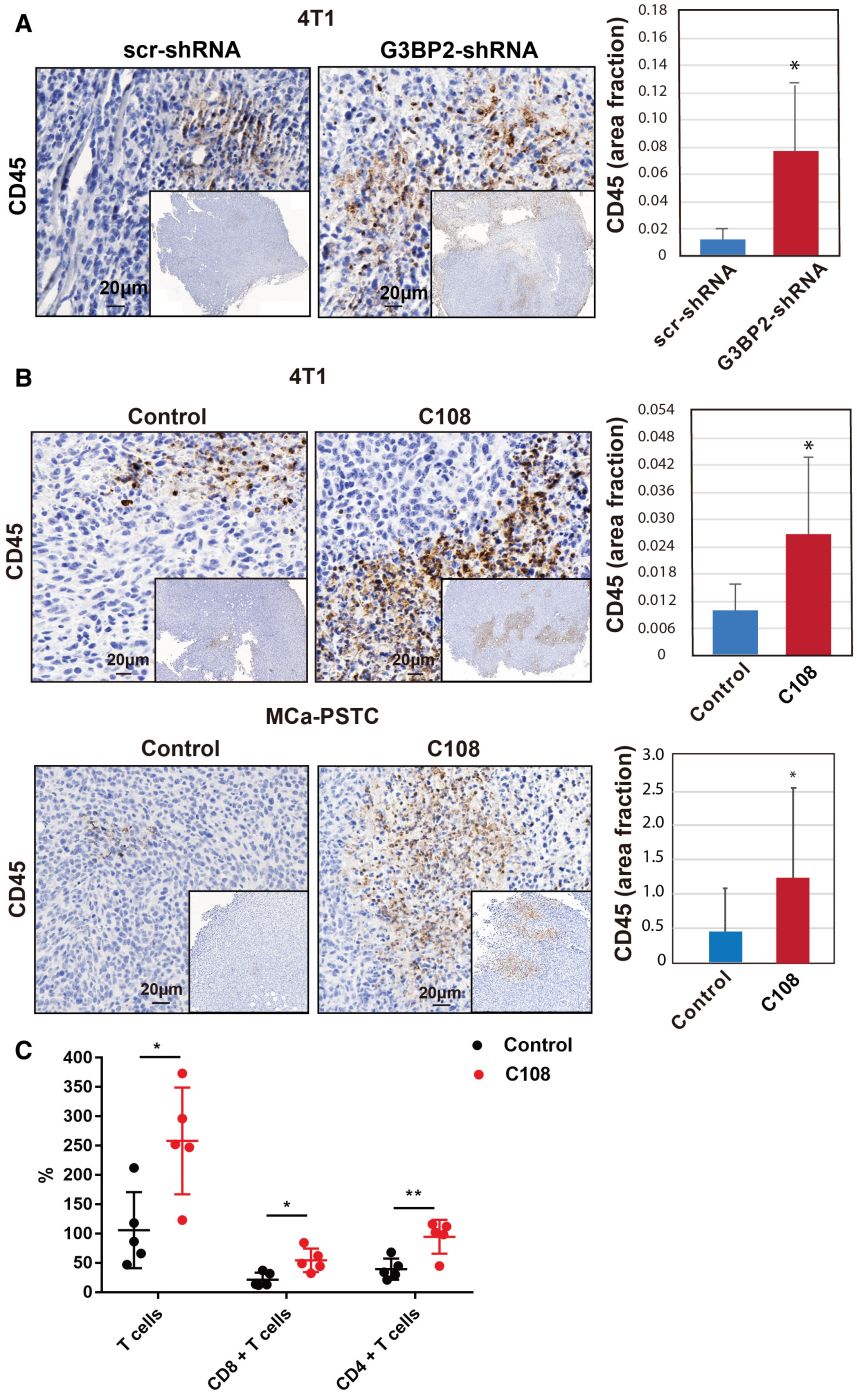
C108 compound increases tumor‐infiltrating hematopoietic‐derived immune cells in primary tumors. (A) Paraffin‐embedded sections from Scr‐shRNA or shG3BP2 4T1 tumors were used for the detection of CD45‐positive cells by immunohistochemistry assay with CD45 antibody (*n* = 9 tumors per group). Data are mean ± SD. (B) Tumor tissues from mice treated with C108 and treated with DMSO were used for immunohistochemistry with CD45 antibodies (*n* = 9, *P < 0.05). Data are mean ± SD. (C) Detection of tumor‐infiltrating CD4+ and CD8+ T cells in treated with C108 compound and untreated breast tumors. Mice were injected with MCa‐PSTC cells in the mammary fat pad of female FVB mice, and the mice were treated with C108 compound (*n* = 5 mice/group). Percentage of cytotoxic tumor‐infiltrating CD4+ and CD8+ T cells and their proliferation status in control and C108‐treated tumors determined by flow cytometry. Data are mean ± SD, and two‐tailed *t*‐tests were used for statistical analysis.

Because G3BP2 inhibition decreased PD‐L1 expression, we next investigated whether the decrease in tumor growth after genetic or C108 suppression of G3BP2 observed in our previous experiment was related to an enhanced antitumor immune response. We first confirmed quantified immune cell populations in the shG3BP2 4T1 tumors by immunohistochemistry. We found that the tumors with depleted G3BP2 had more hematopoietic‐derived immune cells (CD45+) compared with the tumors (Fig. 5A). Similarly, we then treated mice bearing 4T1 and MCa‐PSCT tumors with C108 and showed more immune cell infiltration (CD45+) after treatment with C108 (Fig. 5B). Interestingly, C108 not only increased the infiltration of CD45+ immune cells, but also increased the number of functional cytotoxic tumor‐infiltrating CD8+ T cells and CD4+ T cells in MCa‐PSTC model by flow cytometry (Fig. 5C). Taken together, these data indicate that C108 therapy has the potential to restore antitumor immunity exerted by CTLs and an antitumor effect probably depends on PD‐L1 expression.

### 3.6 G3BP2 binding compound C108 extends animal survival

Encouraged by the *in vivo* results, we next tested whether G3BP2 blockade with C108 would decrease PD‐L1 expression in orthotopic murine tumors and extends animal survival. First, we implanted orthotopically 4T1 cells into mammary fat pad of immunocompetent female BALB/c mice. Next, we randomized animals when tumor diameters reached 3 mm (4T1). Animals were randomized into three different groups treated with (a) control, vehicle‐treated; (b) anti‐PD‐L1 (Bio X Cell, BE0101) (one injection of 200 µg/mice/dose every 3 days); and (c) C108 (30 mg·kg^−1^ twice per day in 50 µL of DMSO). During the treatment of 4T1 tumors, the C108 group had slower tumor growth, resulting in significantly smaller tumors compared with control and anti‐PD‐L1, indicating that C108 is a potent inhibitor of tumor growth.

**Fig. 6:**
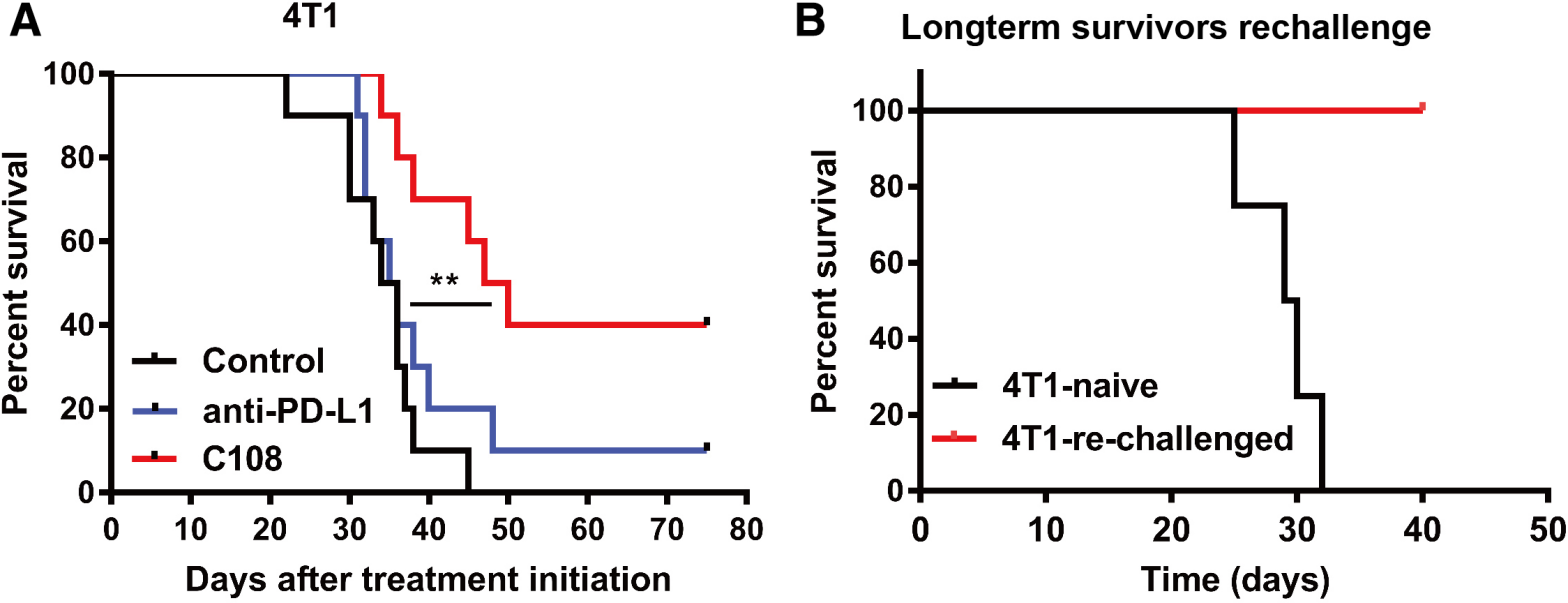
G3BP2 binding compound C108 extends animal survival. (A) The Kaplan–Meier survival analysis for BALB/c mice implanted with 4T1 tumors and treated with C108 (red), anti‐PD‐L1 antibodies (blue), or DMSO (vehicle control, black) (*n* = 10 mice/group, **P < 0.01). The median survival of control, anti‐PD‐L1, and C108 arms was 35, 35.5, and 48.5 days, respectively. C108 treatment resulted in longer survival compared with control (*P* = 0.0009) or anti‐PD‐L1 (*P* = 0.0349); however, anti‐PD‐L1 treatment did not improve median animal survival time compared with control (*P* = 0.2848). Log‐rank test was used for survival analysis. (B) For 4T1 long‐term survivor rechallenge experiment, the control group consisted of age‐matched, treatment‐naïve mice implanted with 1 × 10^4^ 4T1 cells. We resected the primary tumors when the largest tumor reached 10 mm in diameter and observed the survival time in both naïve and rechallenged groups.

We next checked whether C108 would increase survival of animals bearing 4T1 tumors. To approximate the clinical situation in which patients receive neoadjuvant therapy before tumor resection and then follow‐up therapy after surgery, we initiated treatments when the diameter of tumors reached 3 mm. We resected the primary tumors from all mice when the largest tumor dimension reached 10 mm, and continued treatments 10 days postsurgery. We found that monotherapy with anti‐PD‐L1 antibody did not provide a survival benefit, but C108 treatment increased the median survival compared with control or anti‐PD‐L1 antibody groups (Fig. 6A). Moreover, out of 10 4T1 tumor‐bearing mice treated with C108, there were four long‐term survivors, in contrast to only one long‐term survivor in the anti‐PD1 treatment arm (Fig. 6A). In previous work, we found that G3BP2 protein was involved in stimulation of breast tumor‐initiating cells, acting through SART3 and the pluripotency transcription factors Oct‐4 and Nanog [11]. C108 compound directly interacts with G3BP2 protein and inhibits G3BP2 function. Therefore, treatment with C108 compound not only reduced PD‐L1 expression in cancer cells but also inhibited cancer stem cells. This might be the reason for more potent effect of C108 treatment on extending animals' survival, relative to the anti‐PD1 treatment. To test whether mice that survived after C108 treatment had developed long‐term memory, we rechallenged the C108‐treated mice in complete remission by injecting 4T1 cells in the contralateral MFP. We used a new set of age‐matched mice as a control. All treatment‐naïve mice developed progressively growing tumors, while the four previously treated with C108 compound mice rejected this tumor rechallenge (Fig. 6B).

### 3.7 G3BP2 stabilizes PD‐L1 mRNA through a RNA‐binding motif

**Fig. 7:**
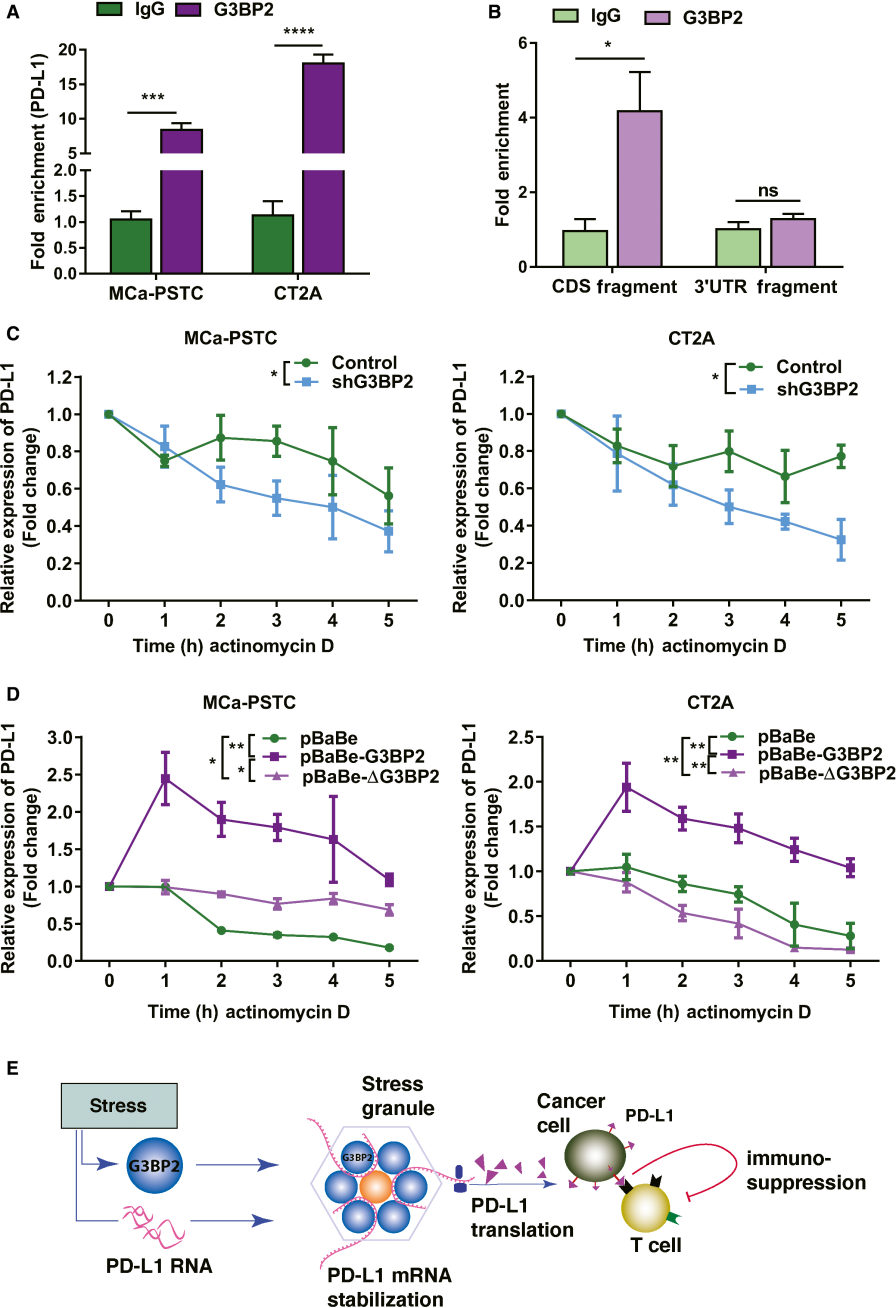
G3BP2 stabilizes PD‐L1 mRNA through a RNA‐binding motif. (A). RIP lysate prepared from MCa‐PSTC and CT2A cells were immunoprecipitated using either a normal rabbit IgG or anti‐G3BP2 antibody, and associated RNA was validated by qRT–PCR using control GAPDH and PD‐L1 primers. Data are mean ± SD of three experiments. Two‐tailed *t*‐tests, ***P < 0.001; ****P < 0.0001. (B) RNA transcripts of the PD‐L1 coding and 3′ untranslated regions were used for RIP assay with subsequent qRT–PCR with PD‐L1 primers. Data are mean ± SD of three experiments. Two‐tailed *t*‐tests, *P < 0.05; ns *P* > 0.05. (C) MCa‐PSTC and CT2A cells with normal and repressed G3BP2 (shG3BP2) were treated with 5 μm actinomycin D for 0, 1, 2, 3, 4, and 5 h. Stability of PD‐L1 mRNA was assessed by qRT–PCR in cancer cells. Data are mean ± SD of three experiments. ANOVA test, *P < 0.05. (D) qRT–PCR was performed to detect PD‐L1 mRNA changes. MCa‐PSTC and CT2A cells infected with pBaBe‐G3BP2 (G3BP2 overexpression), with deletion of RNA‐binding motif of the G3BP2 gene (pBaBe‐ΔG3BP2), and pBaBe as a control were treated with 5 μm actinomycin for 0, 1, 2, 3, 4, and 5 h. The level of PD‐L1 mRNA was assessed by RT–PCR. Data are mean ± SD of three experiments. ANOVA test, *P < 0.05; **P < 0.01. (E) Schematic model of PD‐L1 regulation by G3BP2 in cancer cells.

Since G3BP2 is a multidomain protein that includes an RNA recognition motif (RRM) domain and its RNA high‐affinity binding sequence is tightly associated with a subset of poly(A)^+^ mRNAs, G3BP2 may control mRNA synthesis and degradation through its binding status [8, 9, 10]. To investigate the association of G3BP2 protein with PD‐L1 mRNA, we used RIP. Total lysates prepared from cancer cells were immunoprecipitated using either a normal rabbit IgG or anti‐G3BP2 antibody, and immunoprecipitated PD‐L1 RNA was detected by qPCR with PD‐L1 primers. RIP results demonstrate that G3BP2 protein binds to PD‐L1 mRNA (Fig. 7A). To determine which part of the PD‐L1 mRNA binds to G3BP2 protein, we obtained RNA transcripts from coding and untranslated (3′UTR) regions of PD‐L1 gene (Fig. 7B). Mapping results indicated that G3BP2 protein binds to a coding region of the PD‐L1 mRNA in stressed cancer cells.

We next investigated the mechanism by which G3BP2 modulates PD‐L1. Because G3BP2 is a stress granule protein that sequesters mRNA, we hypothesized that G3BP2 controls PD‐L1 mRNA degradation. Using cell lines with normal or knocked‐down expression of G3BP2, we blocked RNA transcription with actinomycin D (5 μm) and then measured PD‐L1 RNA over time to see its decay. In the MCa‐PSTC and CT2A shG3BP2 cells, there was a substantial decrease in PD‐L1 mRNA stability (Fig. 7C). Inhibition of G3BP2 pharmacologically with C108 resulted in a similar reduction in PD‐L1 mRNA stability (Fig. S2). To confirm that G3BP2 affects PD‐L1 degradation, we cloned the full‐length G3BP2 gene into the pBaBe retrovirus and infected MCa‐PSTC and CT2A cells, which then produced high levels of G3BP2 protein, assessed by western blot and immunostaining (Fig. 2F,G). To test whether the RNA recognition motif region of the G3BP2 protein is crucial for PD‐L1 mRNA stabilization, we also prepared MCa‐PSTC and CT2A cells, which had the RRM sequence of G3BP2 deleted (ΔG3BP2). Consistent with our hypothesis, we found that the overexpression of G3BP2 leads to stabilization of PD‐L1 mRNA and that PD‐L1 mRNA stability was significantly reduced in cells lacking the RRM sequence (Fig. 7D). These results collectively show that G3BP2 stabilizes PD‐L1 mRNA through its RNA‐binding motif (Fig. 7E).

## 4 Discussion

Immune checkpoint inhibitors have revolutionized the treatment of patients with many different types of tumors, but only < 15% of patients benefit from these antibody‐based therapies [19, 20, 21]. In addition to being very expensive, antibodies against the checkpoint molecules can cause severe toxicities [17, 18, 19, 22]. Low molecular weight (MW) drugs are usually less expensive, can penetrate tumor tissues better than antibodies, and can also be orally administrated with appropriate formulation. Since the half‐life of low MW agents is much lower than that of antibodies, the adverse effects of low MW agents can be controlled more rapidly. Tumor cells experience many stresses, including nutrient deprivation from uncontrolled growth and insufficient vasculature. Here, we show that these stresses can directly affect anticancer immunity by upregulating the PD‐L1 checkpoint program. Our attempts to treat cancer impose additional stresses such as chemical toxins and radiation. This link between stress granule formation and immune checkpoint programs provides new opportunities for improving anticancer immunotherapy.

Cancer cells are exposed to many cell‐intrinsic and cell‐extrinsic stresses, and such stresses can directly contribute to tumor progression, immunosuppression, and resistance to various therapies [24, 25, 26]. When exposed to stresses, cells can activate a fundamental, well‐conserved biological program that results in the formation of stress granules (SGs), which limit damage and safeguard important repair machinery such as translation initiation factors, mRNA processing proteins, and ribosomal subunits. In this study, we found that G3BP2, a key SG protein, plays a critical role in regulating PD‐L1 expression in stressed cancer cells.

We also found that immune activation via G3BP2 inhibition is more effective than anti‐PD‐L1 antibody in our animal models. This may be due to more effective immune checkpoint blockade, or because blockade with C108 also interferes with other pathways involved in stem cell production or cancer cell survival. We showed previously that C108 inhibits breast tumor‐initiating cells by repression of the pluripotency transcription factors Oct‐4 and Nanog through SART3 [11]. Based on these studies, inhibition of G3BP2 should have the dual effect of blocking both tumor‐initiating cells and immune checkpoint programs, thus slowing tumor progression and activating antitumor immunity. By identifying the SG forming protein G3BP2 as a common denominator of stemness and immunosuppression, our study provides a compelling mechanism linking these two hallmarks of cancer. This link between stress granule formation and immune checkpoint program also provides new opportunities for improving anticancer immunotherapy.

## 5 Conclusions

In summary, our work has revealed that PD‐L1 level is increased in starved or chemically stressed cancer cells and level of PD‐L1 protein in cancer cells is dependent on G3BP2. Small molecule C108 that binds G3BP2 could decrease PD‐L1 expression due to enhanced mRNA degradation. Moreover, treatment of tumor‐bearing mice with small molecule C108 resulted in increased tumor immune cell infiltration and shows a significant survival benefit and long‐term cures. The potential interplay between stress granule formation and immune checkpoint programs may unveil new targets for anticancer therapy.

## Conflict of interest

The authors declare no conflict of interest.

## Author contributions

YZ acquired the data, analyzed and interpreted the data, and wrote the manuscript. CY acquired the clinical data, and analyzed and interpreted the clinical data. AMK analyzed and interpreted the data, and proof‐read the manuscript. IG conceived and designed the study, acquired the data, analyzed and interpreted the data, and wrote the manuscript. All authors read and approved the final manuscript.

## Supporting information


**Fig. S1.** Expression level of PD‐L1 protein in response to drug stress *in vivo*. Mice were injected with breast cancer cells in the mammary fat pad of female mice and then mice were treated with paclitaxel (10 mg/kg, twice a week). PD‐L1 expression was determines by immunohistochemistry assay. Tumors tissues from treated and untreated with paclitaxel were stained with anti‐PD‐L1 antibodies (brown color). (n = 5 mice/group). Scale bar = 20 µm.

**Fig. S2.** C108 destabilizes PD‐L1 mRNA. A, B. MCa‐PSTC and CT2A cells were treated with 5 μM Actinomycin D for 0, 1, 2 , 3, 4, and 5 hrs or in combination of C108 (4 μM). Stability of PD‐L1 mRNA was assessed by qRT–PCR in cancer cells. Data are mean ± SD of three experiments. ANOVA test, *P < 0.05.

## Data Availability

The raw data are available from the corresponding author upon reasonable request.
