# Supplementary figures and images for: Repression of the stress granule protein G3BP2 inhibits immune checkpoint molecule PD‐L1
Source: Mol Oncol. 2022 Jan 5;19(2):558–71. doi: 10.1002/1878-0261.12915 (PMC11793003; doi:10.1002/1878-0261.12915)

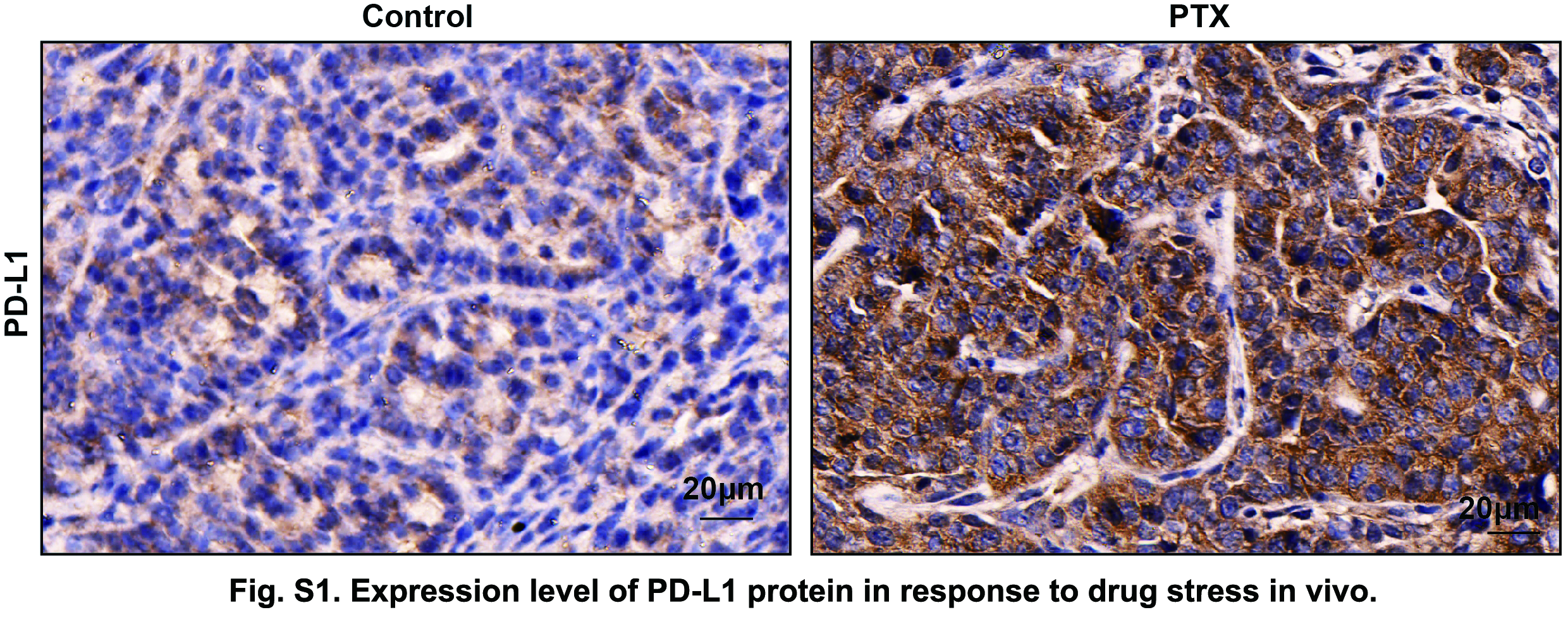

Supplement: Supplementary file 1 — Fig. S1. Expression level of PD‐L1 protein in response to drug stress in vivo. Mice were injected with breast cancer cells in the mammary fat pad of female mice and then mice were treated with paclitaxel (10 mg/kg, twice a week). PD‐L1 expression was determines by immunohistochemistry assay. Tumors tissues from treated and untreated with paclitaxel were stained with anti‐PD‐L1 antibodies (brown color). (n = 5 mice/group). Scale bar = 20 µm. [file MOL2-19-558-s002.tif]

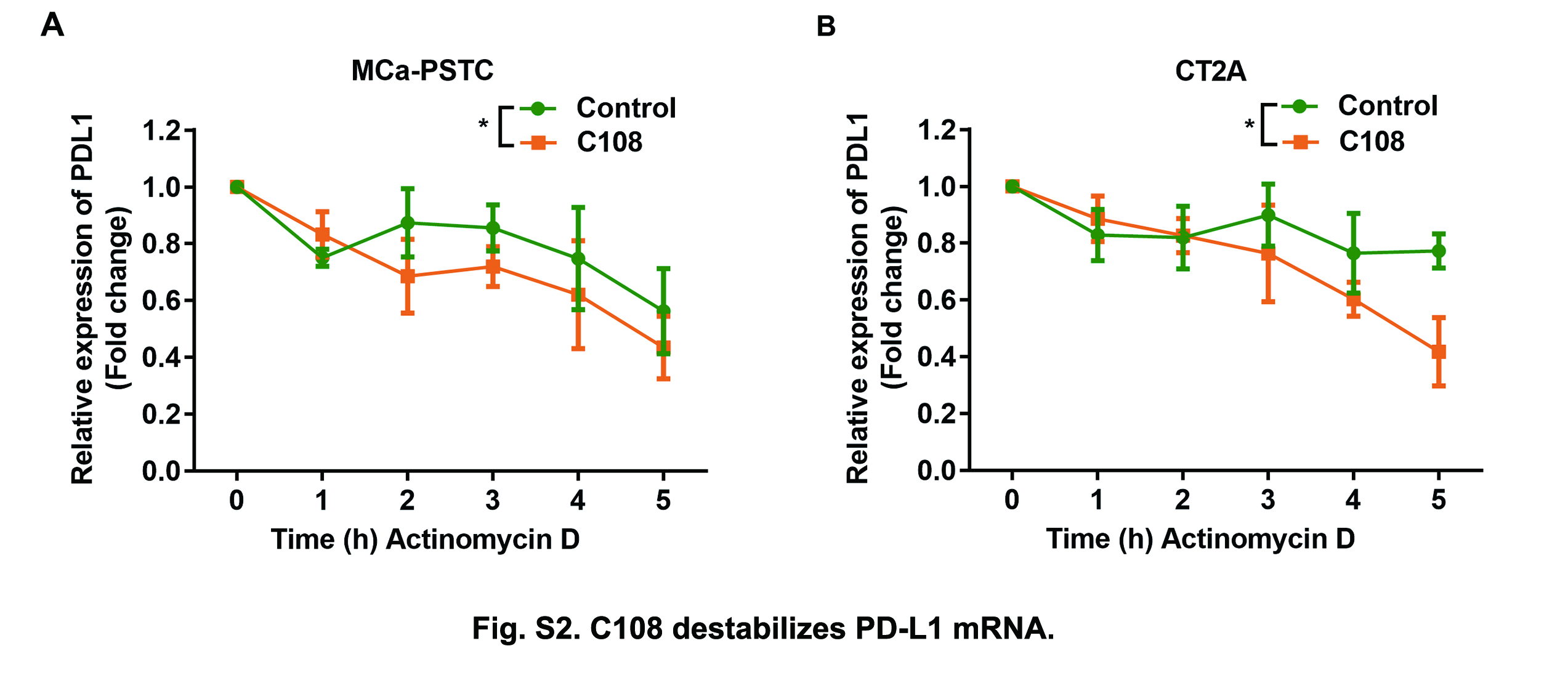

Supplement: Supplementary file 2 — Fig. S2. C108 destabilizes PD‐L1 mRNA. A, B. MCa‐PSTC and CT2A cells were treated with 5 μM Actinomycin D for 0, 1, 2 , 3, 4, and 5 hrs or in combination of C108 (4 μM). Stability of PD‐L1 mRNA was assessed by qRT–PCR in cancer cells. Data are mean ± SD of three experiments. ANOVA test, *P < 0.05. [file MOL2-19-558-s001.tif]
